# Internal Threshold of Toxicological Concern (iTTC): Where We Are Today and What Is Possible in the Near Future

**DOI:** 10.3389/ftox.2020.621541

**Published:** 2021-01-15

**Authors:** Corie A. Ellison, Anne Marie Api, Richard A. Becker, Alina Y. Efremenko, Sanket Gadhia, C. Eric Hack, Nicola J. Hewitt, Mustafa Varcin, Andreas Schepky

**Affiliations:** ^1^The Procter and Gamble Company, Cincinnati, OH, United States; ^2^Research Institute for Fragrance Materials, Woodcliff Lake, NJ, United States; ^3^American Chemistry Council, Washington, DC, United States; ^4^ScitoVation, Limited Liability Company (LLC), Durham, NC, United States; ^5^Cosmetics Europe, Brussels, Belgium; ^6^Beiersdorf AG, Hamburg, Germany

**Keywords:** threshold of toxicological concern, TTC, ITTC, *in vitro* to *in vivo*, IVIVE, physiologically based pharmacokinetic modeling, PBPK, metabolism

## Abstract

The Threshold of Toxicological Concern (TTC) is a risk assessment tool for evaluating low-level exposure to chemicals with limited toxicological data. A next step in the ongoing development of TTC is to extend this concept further so that it can be applied to internal exposures. This refinement of TTC based on plasma concentrations, referred to as internal TTC (iTTC), attempts to convert the chemical-specific external NOAELs (in mg/kg/day) in the TTC database to an estimated internal exposure. A multi-stakeholder collaboration formed, with the aim of establishing an iTTC suitable for human safety risk assessment. Here, we discuss the advances and future directions for the iTTC project, including: (1) results from the systematic literature search for metabolism and pharmacokinetic data for the 1,251 chemicals in the iTTC database; (2) selection of ~350 chemicals that will be included in the final iTTC; (3) an overview of the *in vitro* caco-2 and *in vitro* hepatic metabolism studies currently being generated for the iTTC chemicals; (4) demonstrate how PBPK modeling is being utilized to convert a chemical-specific external NOAEL to an internal exposure; (5) perspective on the next steps in the iTTC project.

## Introduction

Industries and regulatory agencies across the world are challenged with performing human health safety assessments, risk-based prioritizations, and evaluations of thousands of chemicals. *In vivo* testing in animal toxicological studies is time- and resource-intensive, impractical for thousands of substances, and banned for cosmetics ingredients. Therefore, there has been a concerted effort within the scientific and regulatory communities toward the development and utilization of alternative approaches [European Union (EU) Cosmetics Regulation (Regulation 1223/2009); US NRC. US National Research Council, 2017]. The Threshold of Toxicological Concern (TTC) is an approach that can be integrated with knowledge of exposure to enable a protective safety assessment for a single chemical (Kroes et al., 2004) or an efficient risk-based tool for prioritization and screening of thousands of chemicals (Patlewicz et al., 2018). The TTC establishes a low-level exposure value, derived from the evaluation of curated toxicity data from hundreds of chemicals with diverse structures, below which there is a low likelihood of adverse effects for chemicals lacking safety data. If human exposure to a chemical is below the TTC value, it can be judged “with reasonable confidence, to present a low probability of a risk” (Munro et al., 1996). When using TTC for non-cancer endpoints, a tiered approach is utilized rather than applying a single limit value. A chemical is assigned to one of three potency bins based on its chemical structure (Munro et al., 1996). The appropriate potency bins, called “Cramer Classes,” are determined by using the decision tree of Cramer et al. (1978), and are defined as: “Class I substances are those with structures and related data suggesting a low order of oral toxicity”; Class II chemicals are intermediate (less innocuous than Class I); “Class III substances are those that permit no strong initial presumptions of safety, or that may even suggest significant toxicity” (Cramer et al., 1978).

When using the TTC for risk-based evaluations, the relevant TTC value is compared to a human external exposure estimate (e.g., oral intake in mg/kg-day) because TTC values have been derived from toxicological data from oral exposure studies in animals based on administered dose (i.e., mg/kg-day). While TTC has proven to be an important tool for addressing low level exposures, there are times in a safety assessment where the internal exposure is more relevant, and as such, several groups (Bessems et al., 2017; Ellison et al., 2019; Rogiers et al., 2020) have suggested to expand the TTC concept further so that it is representative of internal exposures. This refinement of TTC based on plasma concentrations, referred to as an “internal TTC” (iTTC), attempts to convert external NOAELs in mg/kg/day of chemicals in the TTC database to an estimated corresponding internal exposure. An iTTC would be useful in the development of new approach methods and to further expand and refine the use of TTC. Example uses of the iTTC thresholds include: risk-based safety assessments for dermal and inhalation exposures; development of metabolism-based structure-activity relationship assessments; risk-based screening of aggregate exposures of a given substance from multiple routes of exposures; *in vitro* safety evaluation by comparing the iTTC to concentrations producing bioactivity in *in vitro* biological assays.

**Figure 1:**
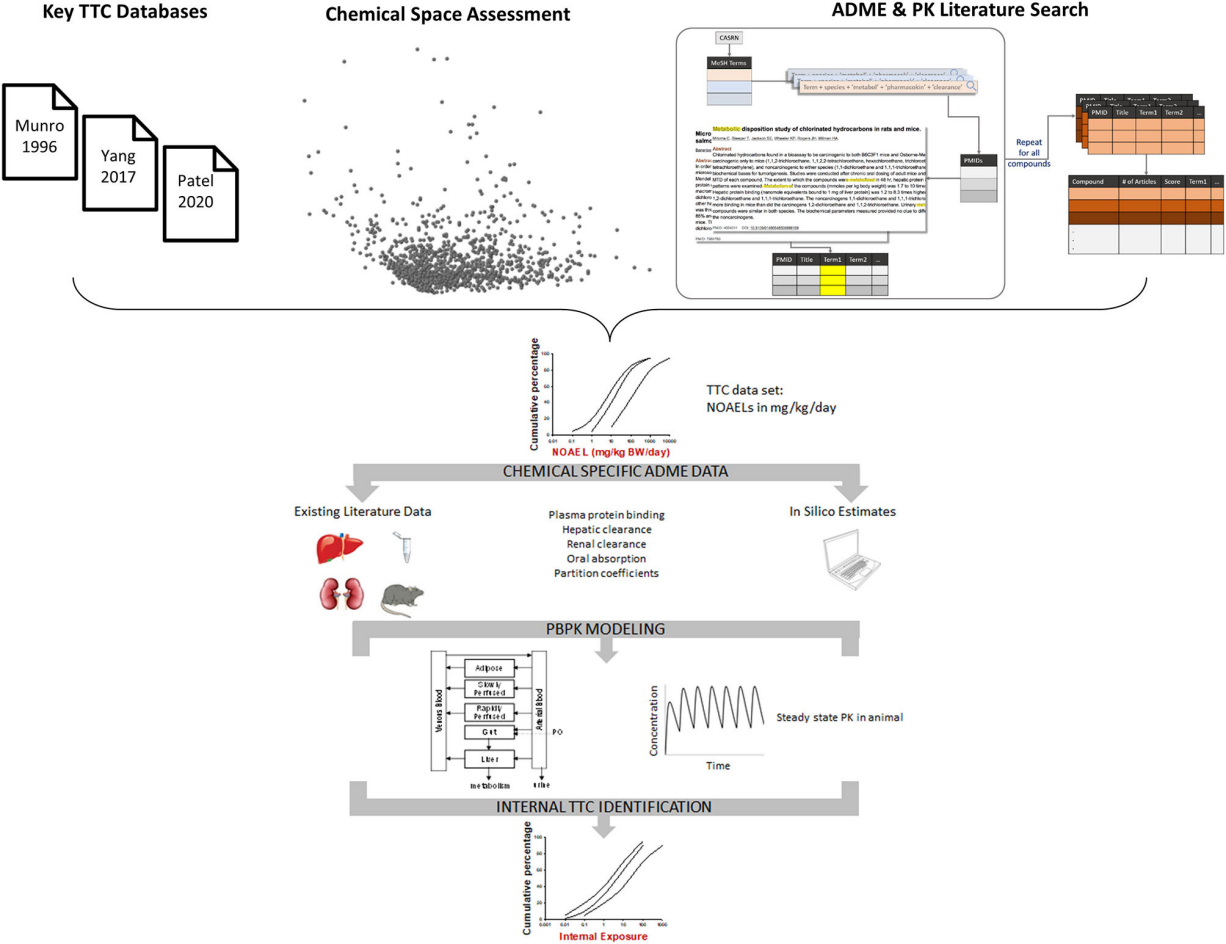
Framework for deriving iTTC values. Chemicals from key TTC databases were combined and the chemical space was mapped using structural and ADME descriptors for the chemicals. Each chemical has undergone a literature search for existing pharmacokinetic (PK) and *in vitro* metabolism data. Results of the chemical space assessment and literature search were used to guide chemical selection for the iTTC project (see Figure 2). The external dose NOAEL for chemicals included in the iTTC project are converted to an internal exposure by using PBPK modeling. The distribution of chemical specific internal exposures is then used to identify iTTC thresholds.

In 2017, Cosmetics Europe (CosEu) organized a workshop with participants from multiple stakeholders (cosmetics and chemical industries, the US EPA, EU JRC, and academia), all with relevant expertise in TTC and Absorption, Distribution, Metabolism, and Excretion (ADME) to critically evaluate the requirements to establish an iTTC (Ellison et al., 2019). At the conclusion of the workshop, a framework and workplan for deriving an iTTC was established (Figure 1). The framework for deriving an iTTC begins with identifying existing TTC/chemical datasets which contain chemical-specific oral NOAELs, expressed as an external dose in mg/kg/day. Then, ADME properties are collected for each chemical in the dataset via existing literature, *in silico* estimation tools, or generating empirical data. Chemical-specific physiologically based pharmacokinetic (PBPK) modeling will then be used to convert the chemical-specific oral NOAELs from the TTC dataset of chemicals to an internal exposure estimate. Multiple advancements have been made toward establishing an iTTC as a result of the multi-stakeholder collaboration. In this manuscript, we discuss the progress and future direction for the iTTC project.

## Literature Search

The iTTC database was generated by combining three existing TTC/chemical databases, including Munro et al. (1996), COSMOS (Yang et al., 2017) and the RIFM database (Patel et al., 2020). The combined database results in ~1,300 unique chemicals. An extensive literature search was conducted for each chemical in the combined chemical database (Munro + COSMOS + RIFM) to identify the chemicals with existing pharmacokinetic (PK) and ADME data. Results from this search were leveraged to prioritize chemicals needing additional data for PBPK modeling, as well as help identify existing *in vivo* data that could be used to verify the PBPK models. The literature search was conducted following the approach described by Ellison et al. (2019) and described in full in Supplemental File 1. In brief, an automated workflow was developed and implemented in Spyder using the Bio-python Entrez package to locate articles in PubMed with the specified species, compound, and either pharmacokinetics, metabolism, or clearance. For each article that was identified by the automated workflow, a text search was conducted to create a term frequency matrix for each article using keywords targeted toward prioritizing papers that have *in vitro* metabolism and/or PK data. The idea was that articles having a higher frequency of specified terms would be more relevant for the project. To rank the importance of a paper, a score was calculated that summed the most important search terms from the list. An extensive quality control check was performed as the data were extracted and collated into three different categories: “*in vitro* data only,” “*in vivo* data only,” and “*in vitro* and *in vivo* data.” Data were curated manually, ensuring the correct species, consistency of clearance units, accuracy of unit conversions, suitability of *in vivo* data, and target exposure routes. The final literature search was conducted for ~1,300 compounds and identified potential published data sources for 603 of the compounds (hit compounds). A total of ~67,800 publications were identified in the search, whereby 312 of the hit compounds had 10 or fewer article hits, while 102 chemicals had over 100 hits. More than 6,000 of the publications were reviewed, with prioritization given to more PK/ADME-relevant publications as described above. At the conclusion of the literature search, it was determined that for the ~1,300 chemicals that were searched, ~80% of the chemicals had no PK or ADME data, 10% had *in vivo* PK data, 5% had *in vitro* metabolism data and 5% had *in vivo* PK and *in vitro* metabolism data.

## Chemical Selection

**Figure 2:**
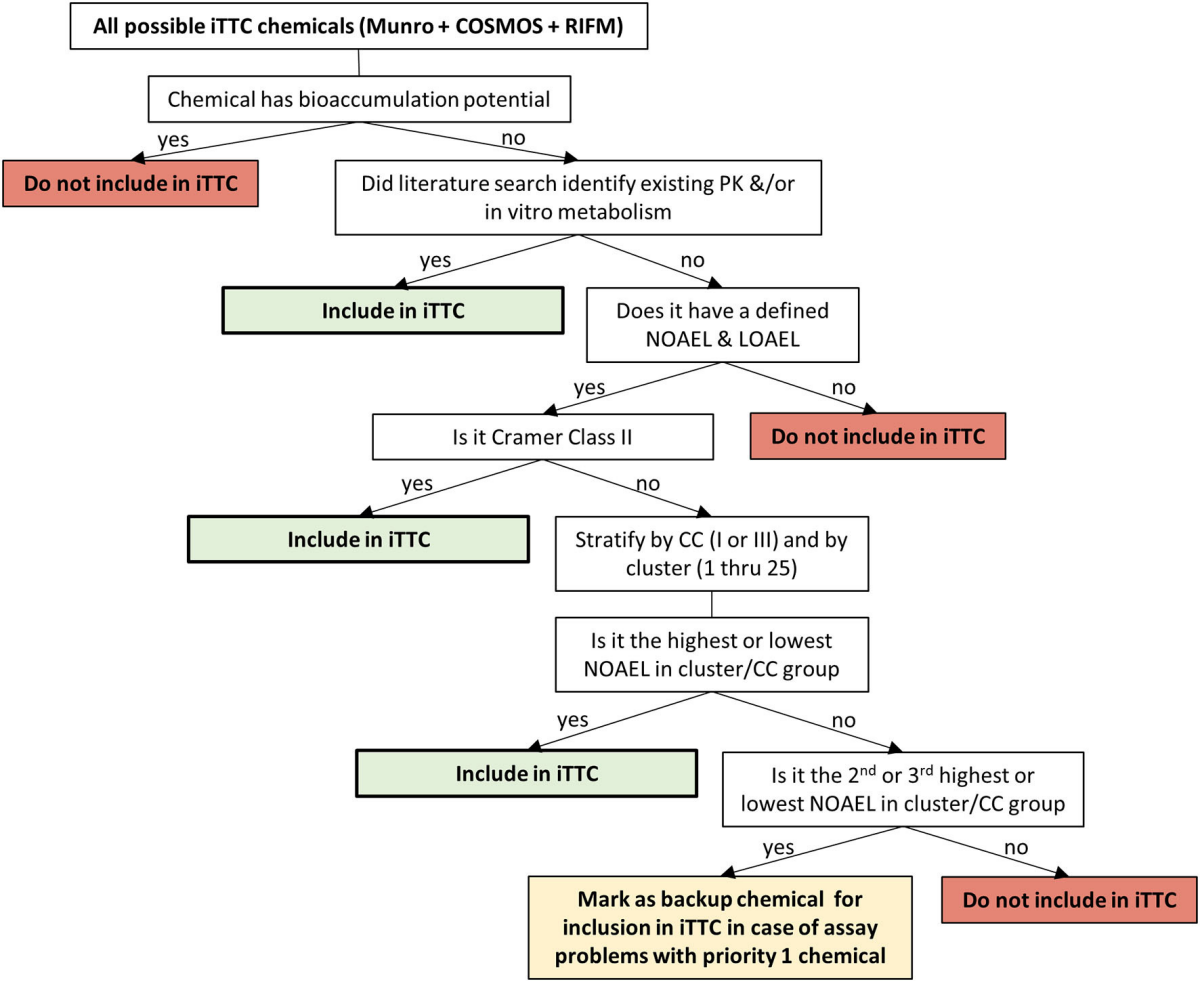
Decision tree for chemical selection for the iTTC project.

The large dataset derived when merging the Munro, COSMOS, and RIFM databases creates a practical challenge for the iTTC project because of the resources needed to generate new *in vitro* ADME data and complete PBPK modeling for such a diverse group of chemicals. Thus, the dataset was triaged to a more manageable number using a chemical space assessment. To complete this, two statistical approaches, principal component analysis (PCA) and k-means clustering were utilized (Ellison et al., 2019). The results from the PCA and k-means clustering analyses helped to separate and group the chemicals so that representative chemicals can be chosen, thus reducing the amount of chemicals requiring PBPK modeling to a more reasonable number while still maintaining chemical diversity. The results of the chemical space assessment reported in Ellison et al. (2019), along with the results of the literature search (described above), were used to guide the chemical selection for the iTTC database (Figure 2).

Criteria for selecting chemicals to be included in the iTTC work consisted of: bioaccumulation potential, availability of PK, or *in vitro* ADME data, presence of a NOAEL and Lowest Observable Adverse Effect Level (LOAEL), Cramer classification, and cluster number (from the statistical approach, *k*-means clustering). Bioaccumulation potential is of interest since plasma concentration can significantly under-predict the total body burden of a chemical that bioaccumulates in tissues. As such, when modeling the plasma concentration at the NOAEL, the PBPK model prediction would be a poor indicator of body burden. Metabolic clearance has been shown to be an important factor in determining bioaccumulation potential (Tonnelier et al., 2012), and polyhalogenated aromatic compounds, such as tetrachlorodibenzodioxin, are known to bioaccumulate and have low metabolism. Therefore, we first identified chemicals that may have bioaccumulation potential by identifying polyhalogenated chemicals. These chemicals were then checked to see if they had been previously classified as having bioaccumulation potential and were listed as persistent organic pollutants by the Stockholm Convention (Stockholm Convention, 2019). For polyhalogenated chemicals not on the Stockholm Convention list of persistent organic pollutants, a thorough review of available animal PK data was conducted to determine the rate of elimination of a chemical from the body. Chemicals with a biological half-life longer than 7 days were considered to have slow elimination and were excluded due to potential bioaccumulation concerns. The review for polyhalogenated chemicals identified 83 out of 1,286 chemicals with more than 2 halogens. Of these 83 chemicals: (1) PK data was available for 78 chemicals; (2) 10 were identified as persistent organic pollutants by the Stockholm Convention; (3) 3 were not identified as persistent organic pollutants by the Stockholm Convention but had biological half-lives >7 days. Based on this review, 13 chemicals were identified as bioaccumulating, and were subsequently excluded from the iTTC project (1,2,4,5-tetrachlorobenzene, chlordane, p,p′-DDT, dicofol, dieldrin, heptachlor, heptachlor epoxide, hexachlorobenzene, hexachlorobutadiene, lindane, mirex, pentachlorophenol, photodieldrin).

To maximize the use of available data, chemicals with existing PK and ADME data were included in the iTTC database. Only chemicals that had a defined NOAEL and LOAEL in the critical toxicity study were considered. The NOAEL used in a TTC database should be the highest dose at which no adverse effects are detected, and it is difficult to be sure that this has been identified unless the dose at which adverse effects begin to appear has also been defined. It would be problematic, and potentially result in artificial exposure limits, if chemicals that do not have a defined LOAEL were to be included. For example, FD&C Blue 1, a common colorant used in food, drugs and cosmetic products, has an oral NOAEL of 200 mg/kg/day, which was the highest dose tested in the study and therefore no LOAEL was identified. Part of the low toxicity potential of FD&C Blue 1 is due to the low oral absorption, which has been estimated to be about 0.3–2% (Brown et al., 1980). If PBPK modeling is attempted for this NOAEL dose it would result in a very low systemic exposure to FD&C Blue 1, due to the low oral absorption. If such a derived internal exposure value were to be included in the iTTC distribution it would artificially lower the distribution and lead to misclassification of FD&C Blue 1 being more potent than it truly is. The remainder of the chemicals were stratified by Cramer classification (I, II, and III) and all the Cramer class II chemicals were included in the iTTC project. The reason for including all Cramer class II compounds at this point was due to the fact that Cramer class II has historically been underrepresented in TTC databases (Patel et al., 2020). Cramer class I and II chemicals were stratified further by cluster numbering (1–25) based on the k-means cluster group number that was assigned to it in Ellison et al. (2019). For each Cramer class/cluster group, the chemicals with the highest and lowest NOAEL from the group were selected to be included in the iTTC database. The rationale for picking the highest and lowest NOAEL from the Cramer class/cluster group is that this would provide a balanced approach to start with and there is no way of knowing *a priori* what the internal exposure associated with a NOAEL will be, since this is controlled by the ADME properties of the chemical. This approach has resulted in a diverse distribution of the TTC chemicals being selected for inclusion in the iTTC project. In total, there are ~300 chemicals included in this work. The number and final selection of chemicals is subject to change based on challenges arising for chemical procurement and analytical feasibility in the *in vitro* assay studies.

## *In vitro* Data Generation

As described in Ellison et al. (2019) and illustrated in Figure 1, it is necessary to generate new *in vitro* data for liver metabolism and/or oral absorption. These data are needed as part of the PBPK modeling approach to convert external NOAELs to estimates of internal plasma concentration. The *in vitro* work is currently on-going and involves determining the permeability of ~300 chemicals in Caco-2 cells and metabolism of ~200 chemicals in hepatocyte suspensions. For the Caco-2 cell assays, bidirectional cell permeability is being measured, with a pH of 6.5 and 7.4 in the apical and basolateral compartment, respectively (to mimic intestinal pH). The first tier of testing with the Caco-2 cells was conducted for a group of “calibration” compounds with known *in situ* single pass intestinal permeability data in rats (Kim et al., 2006; Zakeri-Milani et al., 2007; Lozoya-Agullo et al., 2015). Most of these compounds are not included in the iTTC database but are important compounds for this project because they enable an *in vitro* to *in vivo* correlation to be established for the Caco-2 permeability data. The second tier of Caco-2 cell testing has focused on the iTTC compounds. The *in vitro* to *in vivo* correlation will then be used to estimate *in vivo* permeability for the iTTC compounds that only have Caco-2 permeability data. For metabolism studies, the *in vitro* intrinsic clearance in hepatocytes of test chemicals is being determined using a substrate depletion approach (Obach, 1999). Cryopreserved hepatocytes (isolated from the species from which the *in vivo* toxicity data were generated) are being incubated under physiologically relevant conditions with test compounds at concentrations expected to be well below the Km. Samples are being collected at defined time points up to 2 h and assayed for parent compound content. Intrinsic clearance is being estimated based on the elimination rate constant and normalized to the hepatocyte cell number in the incubation.

## PBPK Modeling

PBPK modeling provides the mechanistic basis for predicting internal concentrations based on external dose using ADME data. In addition to the use of historical animal data, *in vitro* experiments can be used to measure the required PBPK parameters, such as metabolic clearance. Use of these cell-based or subcellular data requires *in vitro* to *in vivo* extrapolation to be relevant for whole animal simulation. PBPK modeling for the iTTC project is being conducted using a custom, batch version of Population Life-course Exposure to Health Effects Model (PLETHEM), which was created specifically to handle a large number of compounds such as in the iTTC database in an efficient manner. The existing version of PLETHEM (Pendse et al., 2020) is designed to be easy to use via a graphical user interface. The PLETHEM platform implements an 11-compartment PBPK model, with built-in physiology for rats and humans. While the majority of the TTC database consists of rat data, a number of NOAELs in the database were from studies in other species (rabbit, mouse, dog, hamster, and monkey), and relevant physiologies are being added to this custom version of PLETHEM to support PBPK modeling for these species. The oral exposure module, the route of interest for iTTC, in the PLETHEM platform handles gavage, drinking water, or diet exposure, and absorption is described using a fraction absorbed (Fa) and a first order absorption rate constant. Fa is a parameter that is not commonly reported in the literature and is a combination of several factors that affect oral absorption. For this project, the oral absorption model in PLETHEM is being redesigned so that Fa is no longer an input parameter. Instead, Fa will be estimated by a mechanistic oral absorption model that uses gastrointestinal transit time and the permeability rate of the chemical through the intestinal wall. Input parameter values for PBPK modeling simulations are a combination of *in silico* and *in vitro* data. *In silico* parameters were estimated using a combination of tools including OPERA, ACD, ADMET Predictor, and GastroPlus (Ellison et al., 2019). These parameters include molecular weight, octanol-water partition coefficient, vapor pressure, water solubility, fraction unbound, and fraction available. *In vitro* input parameters for the model include Caco-2 permeability and metabolism.

## Looking Toward the Future

Work is still in progress for the iTTC project. The studies with hepatocytes and Caco-2 cells are continuing, and the PBPK modeling simulations are being conducted as the data becomes finalized. The PBPK modeling simulations will continue to be run in an iterative process to allow input of new *in vitro* data and to explore the impact of different modeling assumptions and approaches. For example, future modeling work will examine the impact of different clearance mechanisms by comparing model predictions that include or omit plasma protein binding (restricted or unrestricted clearance, respectively). Once all relevant data are available and the PBPK modeling work is completed and a distribution of internal exposures exists, approaches to determine and apply appropriate toxicokinetic and toxicodynamic uncertainty factors will be evaluated and then applied to the 5th percentile threshold level to derive the final iTTC values. Moreover, the knowledge gained with this project will go beyond the development of iTTC and will also be relevant to broader issues that will help advance new approach methods.

## Data Availability Statement

The original contributions presented in the study are included in the article/supplementary material, further inquiries can be directed to the corresponding author/s.

## Author Contributions

AE, CH, and CE contributed to the design and implementation of the literature search. CE, NH, and AS contributed to the selection of iTTC chemicals. RB, AA, SG, NH, and CE contributed to the design and analysis of results for the *in vitro* assays. CH, AE, and CE planned and carried out the PBPK modeling simulations. CE, AA, RB, AE, SG, CH, NH, MV, and AS contributed to the writing of the manuscript. All authors contributed to the article and approved the submitted version.

## Dedication

We would like to express our gratitude and pay our respects to our late colleague, CH who passed away in late September 2020 during the final preparation of this manuscript. He was a dedicated PBPK modeler and instrumental in developing the custom version of the PLETHEM PBPK model for this research project. We dedicate this manuscript to his memory.

## Conflict of Interest

CE was employed by The Procter and Gamble Company, AA and SG were employed by the Research Institute for Fragrance Materials, RB was employed by the American Chemistry Council, AE and CH were employed by ScitoVation LLC, NH and MV were employed by Cosmetic Europe, AS was employed by Beiersdorf AG.

## Supplementary Material

Supplemental File 1 Literature search strategy for iTTC project (word doc).

## Abbreviations

ADME: Absorption, Distribution, Metabolism and Excretion
CosEu: Cosmetics Europe
Fa: Fraction absorbed
iTTC: Internal Threshold of Toxicological Concern
LOAEL: Lowest Observable Adverse Effect Level
NOAEL: No Observable Adverse Effect Levels
PK: Pharmacokinetic
PBPK: Physiologically based pharmacokinetic
PLETHEM: Population Life-course Exposure to Health Effects Model
RIFM: Research Institute for Fragrance Materials
TTC: Threshold of Toxicological Concern.
