# Supplementary material for: Internal Threshold of Toxicological Concern (iTTC): Where We Are Today and What Is Possible in the Near Future
Source: Front Toxicol. 2021 Jan 15;2:621541. doi: 10.3389/ftox.2020.621541 (PMC8915896; doi:10.3389/ftox.2020.621541)
Supplement: Supplemental File 1 — Literature search strategy for iTTC project (word doc). [file Table_1.DOCX]

**Supplemental file 1: Literature search strategy for iTTC project**

A dataset containing toxicity values for approximately 1,300 compounds in the combined COSMOS, Munro, and RIFM databases was used as the source of compounds for the literature review. The dataset features oral toxicological points of departure for six species including rat, rabbit, mouse, dog, hamster, and monkey. Details regarding dosing, species, sex, and subspecies (where available) are also provided.

The basic search strategy was to search the title and abstract of articles in PubMed for a list of keywords related to *in vitro* and *in vivo* kinetics. The search was limited to data that matched the species and route of exposure used for each compound point of departure (POD) in the database. A feasible scope of review was established by defining the types of data to be extracted from the articles. Target *in vitro* data includes Vmax and Km, or intrinsic clearance measured in liver microsomes, cytosol, S9, hepatocytes, slices, or homogenates. Target *in vivo* data includes blood, serum, or plasma concentrations, or non-compartmental analysis parameters (e.g., blood AUC, C_max_, T_max_) following oral or intravenous administration. Data from radiolabeled compound studies with no separation of parent and metabolites (i.e., total radioactivity only) were not generally collected. Identified target data were extracted from the publications and compiled in Excel spreadsheets.

A ‘flat’ search of keywords simultaneously was found to lack focus, resulting in excessive numbers of literature hits, many of which were missing the primary information (i.e., *in vitro* clearance and *in vivo* pharmacokinetic data). A tiered search strategy was devised to focus the search. First, a search was conducted for compound name and synonyms, species, and metabolism or pharmacokinetics or clearance. The request submitted to PubMed was:({compound/synonym}) AND ("{species}" OR "-{species}" OR "{species_plural}") AND (metabolism OR pharmacokinetic* OR clearance). The search was performed with respect to a specific species in which POD data was collected and its plural counterpart (ex. “mouse” and “mice”). A secondary search for a broader set of keywords was performed on the results from the primary PubMed search using the following keywords: “absor”, “in vivo”, “in vitro”, “cell line”, “animal”, “incubation”, “intestinal”, “fraction unbound”, “fub”, “Cmax”, “C(max)”, “Tmax”, “T(max)”, “AUC”, “Vmax”, “V(max)”, “Km”, “oral”, “fraction absorbed”, “urinary excretion”, “drug solubility”, “drug permeability”, “colon”, “small intestine”, “permeability”, “blood”, “serum”, “plasma”, “Sprague-Dawley”, “Fischer 344”, “Wistar”, “caco-2”, “hepatocytes”, “S9”, “microsomes”, “protein binding”, “IVIVE”, “Css”, “hydroly”, “excret”, “bioavailab”, “efflux”, “influx”, “active uptake”, “active transport”, “half-life”, “cell”, and MeSH terms. The keywords used included truncated versions of “metabolism”, “pharmacokinetics”, “clearance”, “absorption”, etc. Truncating the words enabled more flexibility in the secondary term search, for example, searching “metabol” was able to match to “metabolism”, “metabolic”, “metabolite”, and etc.

One-compound-at-a-time literature searches were too inefficient, and an automated workflow was developed to conduct a high throughput search of the literature for each compound (Figure 1). This workflow was implemented in Spyder using the Bio-python Entrez package to find articles with the specified compound, species, and either metabolism, pharmacokinetics, or clearance in PubMed. Since the compounds often had multiple names, a list of synonyms was curated for each compound by searching the Medical Subject Heading (MeSH) database with its CASRN, and PubMed was searched iteratively using each compound’s name and list of synonyms.

**
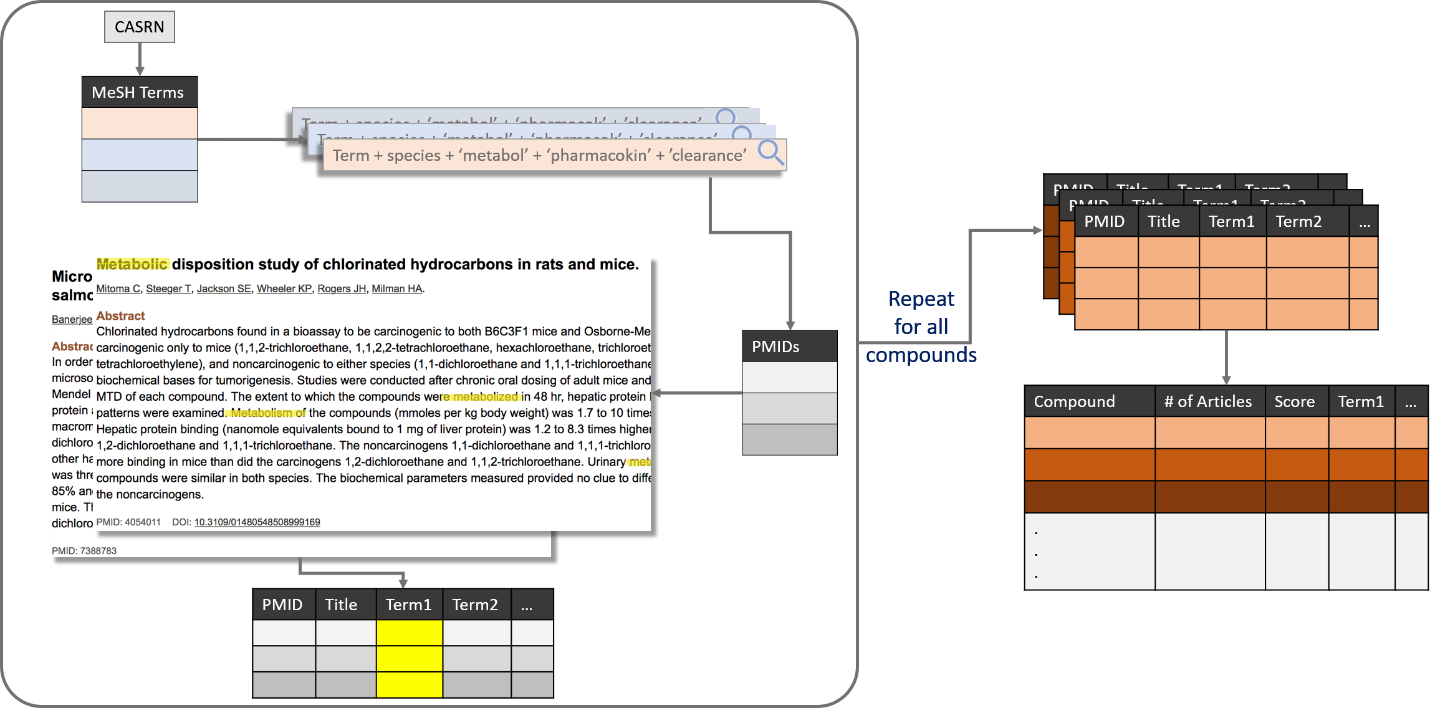
**

**Figure 1.** Workflow of finding articles with data for IVIVE modeling for a large set of compounds

Because of abstract formatting issues using Bio-python Entrez, some abstracts could not be pulled. The affected articles were searched using a supplementary script that scraped the html code of the respective articles’ PubMed page for the terms.

Even with the tiered search, an extremely large volume of publications for review was identified. A scoring metric was developed to prioritize the articles for download and review. A search term frequency matrix was developed containing the number of times each keyword appeared in the title and abstract of each publication. A total score was calculated for each publication, consisting of the sum of a metabolism score designed to indicate the likelihood of *in vitro* clearance data, and a pharmacokinetic score for *in vivo* data. The metabolism score was the sum of the frequency of appearance of “metabol”, “clearance”, ‘hepatocytes”, “S9”, “microsomes”, “Vmax”, “Km”, “hydroly”, and “half-life”. The pharmacokinetic score is the sum of the frequencies for “pharmacokin”, “Cmax”, “Tmax”, “AUC”, “Css”, “blood”, “serum”, and “plasma”. The scores were used to prioritize articles for review, assuming the publications with higher scores were more likely to contain relevant data.

The review of literature search results was conducted in two phases to maximize efficiency. In phase 1, the top 10 scoring publications were reviewed for each chemical, extracting any target *in vitro* and *in vivo* data. If no data were found in phase 1, the articles were rescored by the PK score alone to focus on identifying *in vivo* data in phase 2, and the top 20 articles were reviewed. This was decided since additional *in vivo* data cannot be generated, while new *in vitro* data can be generated. Chemicals were assigned to “in vitro”, “in vivo”, “in vitro and in vivo”, or “no data” bins.

An extensive quality control check was performed as the data were extracted and collated into databases for *in vitro*, *in vivo*, and *in vitro* & *in vivo* data. Data were curated manually, ensuring the correct species, consistency of clearance units, accuracy of unit conversions, suitability of *in vivo* data, target exposure routes, etc. Clearance units were converted to the units used by Population Life-course Exposure to Health Effects Model (PLETHEM) (Table 1). The target *in vivo* data were considered suitable for inclusion if they were oral or intravenous exposure, in the animal species in question, in the blood or plasma, and the parent data were determined separately from the metabolites (e.g., not total radioactivity). The data were compiled into 3 databases: *in vitro* data, *in vivo* data, and *in vitro* & *in vivo* data.

Table 1. Final clearance units for the PLETHEM batch file.

| Source Type | Units |
| --- | --- |
| Microsomes | µL/min/mg protein |
| Cytosol | µL/min/mg protein |
| Hepatocytes | L/hr/million cells |
| Liver slices | L/hr/kg liver |
| Liver homogenates | L/hr/kg liver |

The majority of the *in vitro* data that were extracted were reported as a clearance rate, or as Vmax and Km. In many publications, only an *in vitro* reaction velocity was reported, without Km. In some of these cases, when the substrate concentration was reported along with the velocity, and the substrate concentration was not above micromolar range, it was assumed that the substrate concentration used was Km. These values are flagged in the results, as they have higher uncertainty than the values with a Km or intrinsic clearance determined.
